# The Twitter of Babel: Mapping World Languages through Microblogging Platforms

**DOI:** 10.1371/journal.pone.0061981

**Published:** 2013-04-18

**Authors:** Delia Mocanu, Andrea Baronchelli, Nicola Perra, Bruno Gonçalves, Qian Zhang, Alessandro Vespignani

**Affiliations:** 1 Laboratory for the Modeling of Biological and Socio-technical Systems, Northeastern University, Boston, Massachusetts, United States of America; 2 Aix Marseille Université, CNRS, CPT, UMR 7332, Marseille, France; 3 Institute for Quantitative Social Sciences at Harvard University, Cambridge, Massachusetts, United States of America; 4 Institute for Scientific Interchange Foundation, Turin, Italy; University of Zaragoza, Spain

## Abstract

Large scale analysis and statistics of socio-technical systems that just a few short years ago would have required the use of consistent economic and human resources can nowadays be conveniently performed by mining the enormous amount of digital data produced by human activities. Although a characterization of several aspects of our societies is emerging from the data revolution, a number of questions concerning the reliability and the biases inherent to the big data “proxies” of social life are still open. Here, we survey worldwide linguistic indicators and trends through the analysis of a large-scale dataset of microblogging posts. We show that available data allow for the study of language geography at scales ranging from country-level aggregation to specific city neighborhoods. The high resolution and coverage of the data allows us to investigate different indicators such as the linguistic homogeneity of different countries, the touristic seasonal patterns within countries and the geographical distribution of different languages in multilingual regions. This work highlights the potential of geolocalized studies of open data sources to improve current analysis and develop indicators for major social phenomena in specific communities.

## Introduction

Modern life, with its increasing reliance on digital technologies, is opening unanticipated opportunities for the study of human behavior and large scale societal trends. Cell phones have been playing a pivotal role in this revolution, serving as ubiquitous sensors, and the default point of contact for online activities [1], [2]. As a whole, mobile clients for microblogging platforms, social networking tools, and other “proxy” data of human activity collected in the web allow for the quantitative analysis of social systems at a scale that would have been unimaginable just a few years ago [3]–[6]. In particular, the possibility of using mobile-enabled microblogging platforms, such as Twitter, as monitors of public opinion and social movements and as tools for the mapping of social communities has generated much interest in the literature [7]–[14]. At the same time it is crucial to understand to which extent the picture of socio-technical systems emerging from digital data proxies is statistically sound and how well it does scale to a planetary dimension [15].

In this paper, we perform a comprehensive survey of the worldwide linguistic landscape as emerging from mining the Twitter microblogging platform. Our large-scale dataset, gathered over approximately two years, at an average rate of 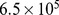 *GPS-tagged* tweets per day, contains information about almost 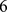 million users and provides a uniquely fine-grained survey of worldwide linguistic trends. By coupling the geographical layer to the identification of the language of single tweets we are able to determine the detailed language geography of more than 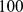 countries worldwide [16].

Although previous studies have investigated the language dynamics of Twitter [17], those analyses have focused on specific, yet interesting, aspects concerning the combined study of language and geographical analysis in Twitter, and a global picture is still lacking. For instance, most represented languages have been identified for the Top-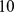 more active countries [18], language-dependent differences have been pointed out in the user activity related to the posting and conversations patterns [19], and language has been shown to be a strong predictor for the formation of follower/followee relations [20]. For this reason and for the sake of assessing the generality and planetary scalability of our analysis, we have first focused on the reliability of geospatial trends extracted from our dataset. Interestingly, we find a universal pattern describing users' activity across countries, and a clear correlation between Twitter adoption and the Gross Domestic Product (GDP) of a country, further characterized by well defined continent-dependent trends.

The high quality of the dataset permits the study of the spatial distribution of different languages at different scales from aggregated country-level analysis to the neighborhood scale. In particular we can drill down data of linguistic macro areas and single out heterogeneities at the country and regional level, scrutinizing the cases offered from Belgium and Catalonia (Spain) as examples. Furthermore we explore the resolution offered by the data at very fine level of granularity and inspect the city and neighborhood levels, taking as case studies the spatial distribution of French and English languages in Montreal (Canada) and inspecting linguistic majorities in New York City (USA). We find that Twitter is able to reproduce the geospatial adoption of languages for a wide range of resolution scales. Finally, we broaden our perspective by addressing the seasonality patterns in the language composition of the Twitter signal. We use touristic countries such as Italy, Spain, and France to single out clear seasonal trends like, for instance, the increase of English and other languages during the summer holiday season. Overall, our analysis highlights the potential of Twitter data in defining open source indicators for geospatial trends at the planetary scale. Although we focus on specific examples of the Twitter language use at different geographic resolutions, our analysis has been performed worldwide and specific areas of the world may be investigated by using the data exploratory that we have made available to the research community (http://www.mobs-lab.org/language.html).

The paper is structured as follows. In the Results section we go over data selection criteria as well as statistical measures regarding the universality of users behavior. Within this framework, we investigate several relevant examples in language geography and explore the temporal dimension for seasonal patterns. A discussion of the results is followed by a thorough description of the data sets and methodology used.

## Results

Our analysis is based upon Twitter data gathered in approximately 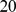 months between October 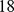, 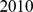 and May 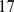, 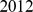, at an average rate of 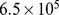 GPS-tagged tweets per day (see Table 1 for exact numbers). The dataset includes 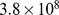 tweets produced by 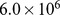 users located in 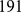 countries, 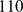 of which generated the amount of data necessary for a significant statistical analysis of language detection. Our language detection methods allowed us to identify 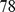 languages. Our analysis is restricted to GPS-tagged tweets in order to preserve maximum level of geographical detail, taking into account both live GPS updates and device stored locations. The amount of geolocalized signal could in fact be increased by considering different kinds of metadata, like for example self reported locations [13], but these procedures would not allow us to reach the level of granularity and detail we aim to. Further details about the data collection and analysis procedures, as well as on the (live) GPS metadata, can be found in the Methods section. Overall, considering the recent literature, and to the best of our knowledge, the amount of GPS-tagged data we have gathered is certainly remarkable not only in terms of volume, but also for the covered geographical and temporal extension.

**Table 1:** Basic metrics of the data set.

Days of data collection	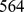
Tweets/day GPS (live-GPS)	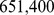 ( 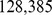 )
Users (users live-GPS)	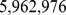 ( 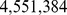 )
Countries (total)	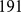
Countries (analyzed)	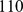
Detected languages	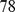

Along with the total GPS signal, the fraction of live updates is reported (see Methods for details).

**Figure 1:**
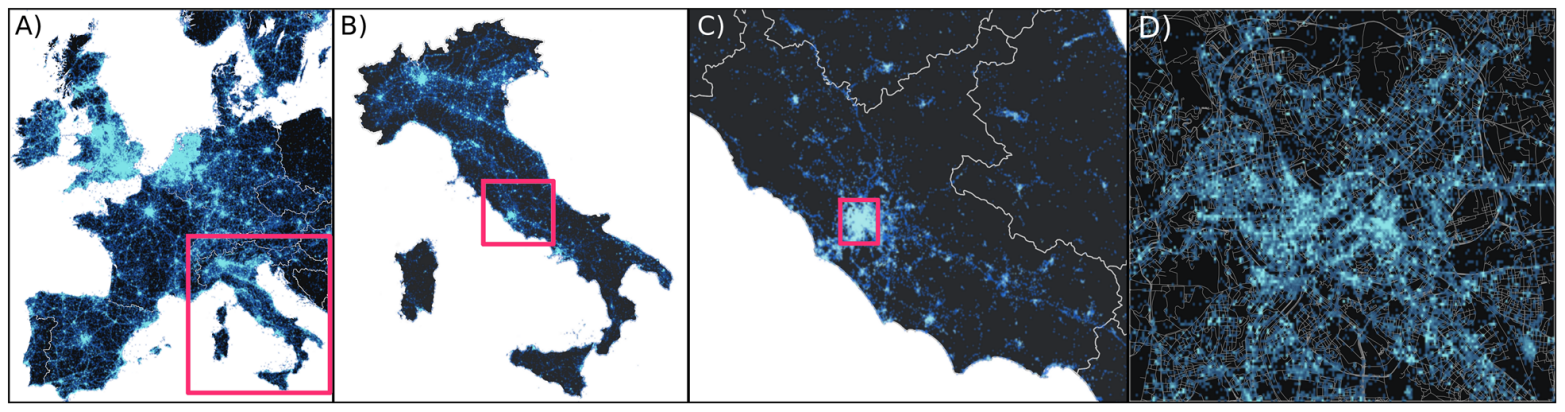
Multiscale view of the geolocated Twitter signal. The large number of geolocated Twitter traffic allows for a high resolution characterization of human behavior. A) Europe B) Italy C) Lazio region D) Rome. The squares highlight the zooming areas.

**Figure 2:**
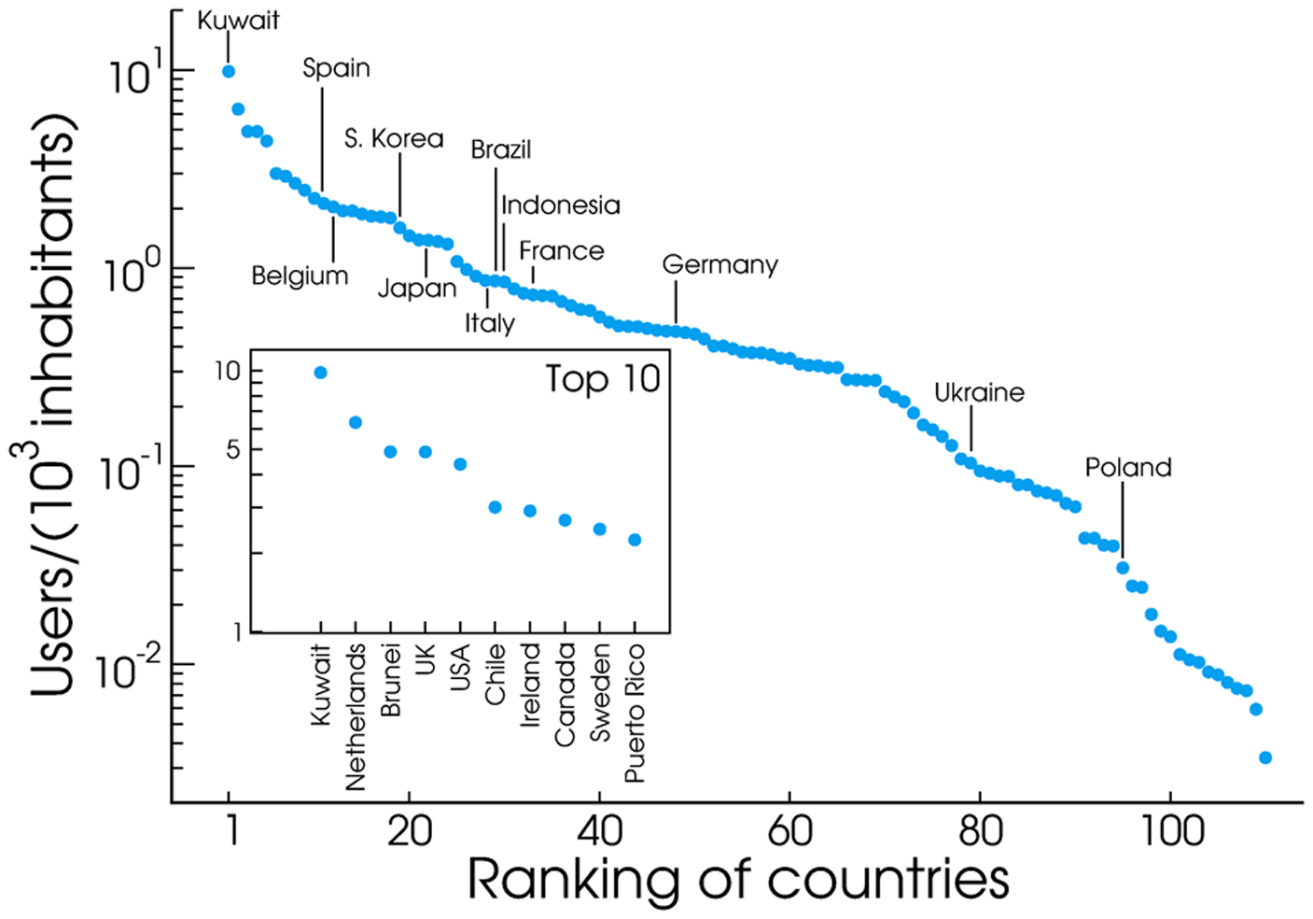
Ranking of countries by users per capita. Ranking of countries as per average number of Twitter users over a population of 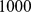 individuals.

**Figure 3:**
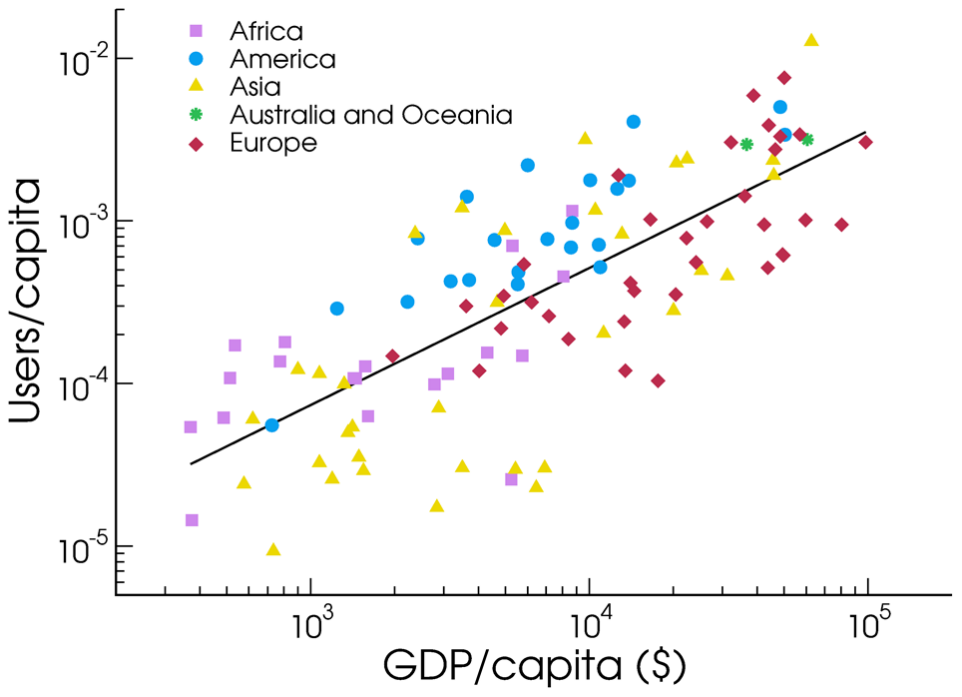
Users and GDP per capita. Correlation between country level Twitter penetration and GDP/capita. The adjusted R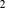 value of the fit is 0.56.

Fig. 1 illustrates the potential of inspection at different resolutions, from continent to city level, highlighting the detailed structure that is visible at each scale. Countries are easily identified along with their major metropolitan areas, and even within specific cities it is possible to observe a high degree of details. Coupling this geographical resolution with language detection tools (see Methods) provides us with a remarkable view of how languages are used in different areas. However, Twitter adoption is not homogeneous across different countries. Fig. 2 ranks countries in descending order in terms of Twitter adoption, defined as the ratio between Twitter users and total population (i.e. Twitter users per 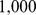 inhabitants). The emerging picture is highly heterogeneous, as expected, since our data come exclusively from smartphone devices that are consequentially tied to the availability of local infrastructures. In order to support the hypothesis that economic diversity is a primary source of heterogeneity in the Twitter adoption (in mobile devices), we investigated whether the Gross Domestic Product (GDP) of a country could serve as a predictor of microblogging adoption. Fig. 3 shows that this is the case, the GDP and the Twitter users per capita being clearly correlated. Moreover, different continents (identified by different color codes in Fig. 3) cluster together, with African and Asian countries occupying mostly the bottom left portion of the graph, Europe and Oceania the top right corner and America the intermediate region. Of course exceptions are present, but this trend indicates that cultural as well as socio-economic factors concur at once in determining the observed pattern.

**Figure 4:**
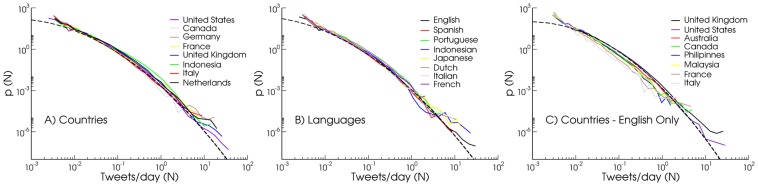
User Activity. Probability density 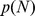 of user activity (number of daily tweets N) grouped by country (A) and language (B), and by country while considering English tweets exclusively (C). Different curves collapse naturally, without any functional rescaling, indicating the presence of a seemingly universal distribution of users activity, independent from cultural backgrounds. Countries in panel (A) and (C) are characterized by high Twitter penetration and represent different continents, while the languages in panel (B) are selected from those producing very strong signal. Dashed lines represent log-normal distributions 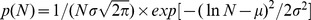, with 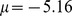 and 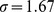 for (A), 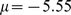 and 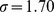 (B), and 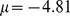 and 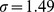 (C).

Geographical analyses at any scale require the aggregation of the signal produced by different users, and it is crucial to have a clear understanding of the patterns of single user activity. One might suspect that usage patterns at the individual level may show large heterogeneities across country and thus cultures. In order to test statistically the presence of different usage patterns we gather the number of tweets per unit time sent by each single identified user. From this data we construct the probability density function 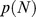 that any given user emits 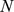 tweets per considered unit time. In our analysis we considered as reference unit time one day. Furthermore, the 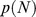 distribution can be analyzed by restricting the statistical analysis to users belonging to a specific country, a specific language or both. Interestingly, Fig. 4 shows that the distributions exhibit a universal shape irrespective both of country (panel A), of language (panel B), or of the weight of each countries on a specific language (panel C). As we will see this finding is pivotal for an unbiased comparison of different geographical and linguistic scenarios. Any dependence of the activity distribution upon the language or location of the users would have reduced the array of possible analysis. It is worth stressing also that the curves overlap each other naturally, i.e., with no need for any rescaling or transformation. This universal behavior is well fitted by a log-normal distribution as shown in Figure 4, and confirmed by the Shapiro-Wilk test (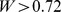 for all languages but Italian (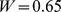) in panel A, 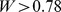 for all curves in panel B, and 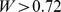 for curves in panel C (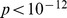)). Although the universality of the users' behavior indicates a very strong statistical homogeneity at the population level, the observed distribution turns out to span almost 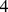 orders of magnitude. The broad nature of this universal distribution is clear evidence of strong individual level heterogeneity. For this reason, in order to avoid distortions due to extremely active users, we consider only the proportion of tweets emitted by each user in a given language. Thus, a user 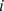 that tweets in a set, 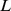, of different languages, 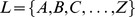, will contribute to each language 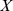 for a fraction 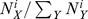. We define 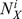 the total number of tweets written by the user in language 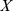. We adopt the same normalization also for the position of the user. The reasons for this normalization are multiple. First, the amount of tweets collected for each user ranges over several orders of magnitude. Very active users, as well as automatic bots, might therefore distort or mask the signal coming from “common” individuals. Second, tourism might be a strong source of noise when trying to understand the demographics of a country or of a city. Touristic locations in the South of France or Italy might, for example, exhibit a high proportion of tweets in English or German. It is worth noting that our method takes into account also users with low activity, since we consider that they represent a significant signal when language distribution is considered. For different analysis, however, they might represent a source of noise, and a threshold on minimum activity could be useful.

### Language analysis at different geographic scales

**Figure 5:**
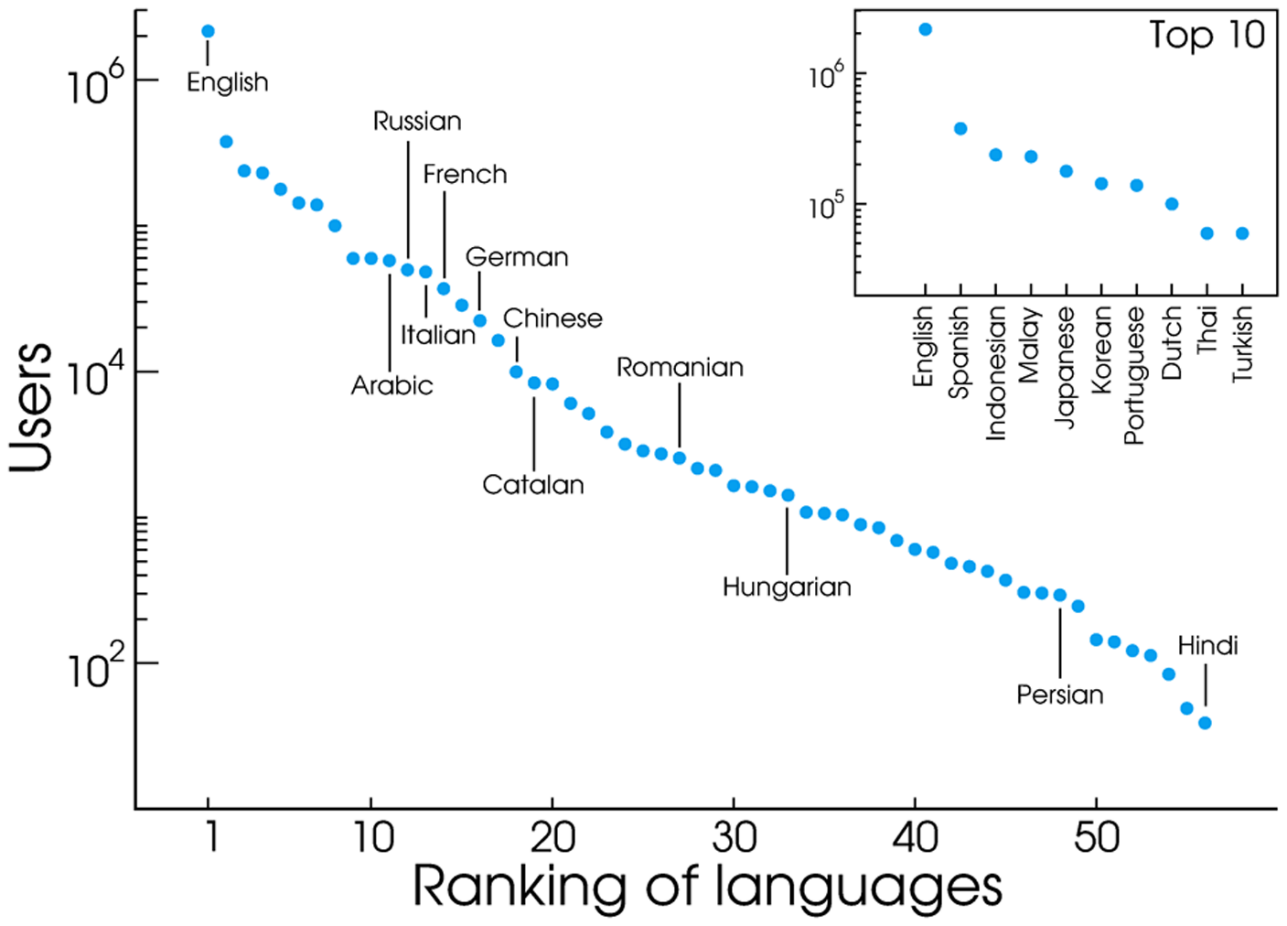
Languages by number of users. Languages ranked by total number of users. For clarity, only languages with more than 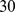 users are shown.

**Figure 6:**
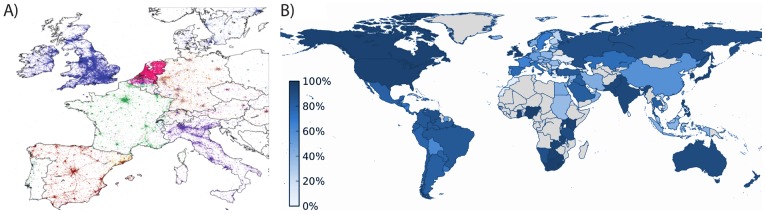
Geographic distribution of languages around the world. A) Raw Twitter signal. Each color corresponds to a language. Densely populated areas are easily identified, while, as expected, languages are well separated among European countries. B) Dominant language usage. The color of each country indicates the fraction of users adopting the official language in tweets. Gray represent countries without statistically significant signal.

The ranking of languages in our signal is presented in Fig. 5, where the ordering is determined by the number of users we observe for each one of them. As expected, English is largely dominant. Spanish occupies the second position despite being almost 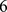 times less popular. Interestingly, these languages are followed by Malay and Indonesian, reflecting the fact that Indonesia is a very active country in absolute terms, even though in terms of users per capita the country is only ranked in the 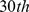 position (see Fig. 6). Here, the effect of each country's population size becomes clear. A large country as Indonesia does not need a large per capita Twitter penetration to make its language very visible in Twitter, while much smaller Netherlands does. And in fact the Netherlands is the second country in terms of users per capita (see Fig. 6), making Dutch the 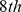 most common language.

It is worth stressing that our statistics *do not* reflect the overall estimates of language speakers in the world. According to Ethnologue: Languages of the World [21] (as aggregated in [22], where different statistics are also reported), when native and secondary speakers are considered together Standard Chinese leads the ranking (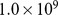 speakers), followed by English (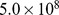 speakers), Spanish (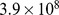 speakers), Hindi (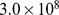 speakers) and Russian (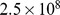 speakers), with Malay/Indonesian ranked as 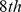 (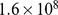 speakers). These discrepancies do not prevent us from extracting meaningful information in countries where Twitter is sufficiently high to serve as an accurate mirror of the population, but it serves as a reminder that we are observing the worldwide linguistic landscape through the lenses of a (specific) microblogging platform which, for example, is not available in China. Also the age and census composition of Twitter users must be taken into account if one is to compensate for differences with respect to the official census data [23].

**Figure 7:**
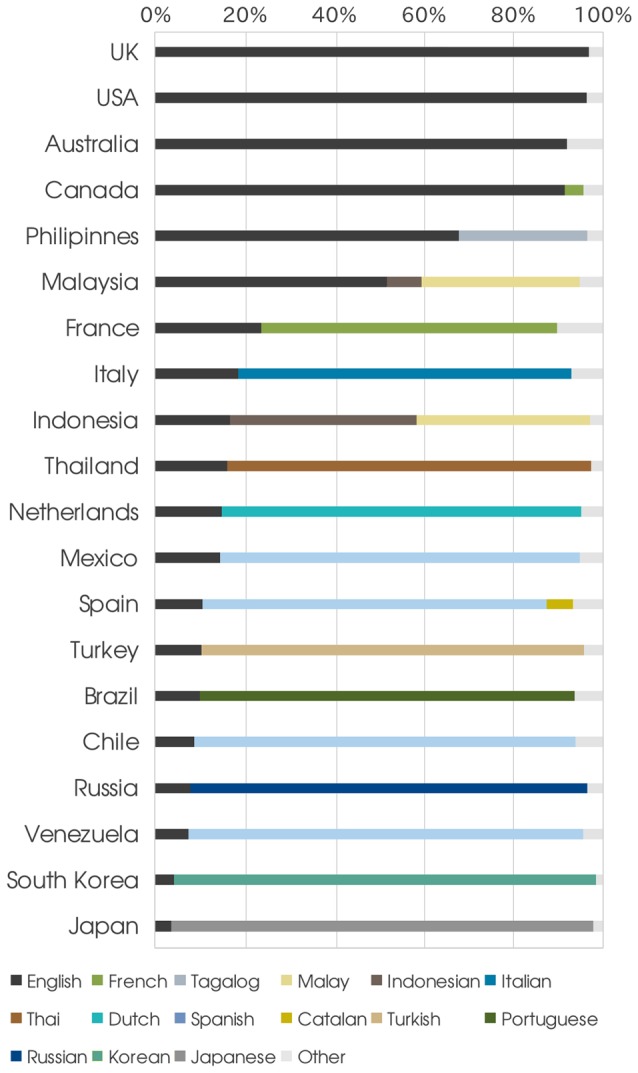
Language share of the most active countries. Language adopted by users coming from Top 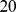 most active countries, ordered by number of English tweets.

**Country level**. When we color each tweet according to its language and display them on a map we see immediately that most content produced within each country is written in its own dominant language (see Fig 6-A). This is further confirmed in Fig 6-B, which shows the extent to which the dominant language prevails over other idioms in each country. In Figure 7 we plot, for each of the Top 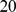 countries (by number of tweets), the fraction of users tweeting in each language. Interestingly, countries like France and Italy, which are characterized by a well defined and substantially homogeneous linguistic identity, emit more than 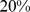 of their tweets in English and other languages. Since the most common language in Twitter is English, this is perhaps not surprising. It is in fact reasonable that even users of non-English speaking countries choose to Tweet in English as a form of reaching out to a broader audience.

**Figure 8:**
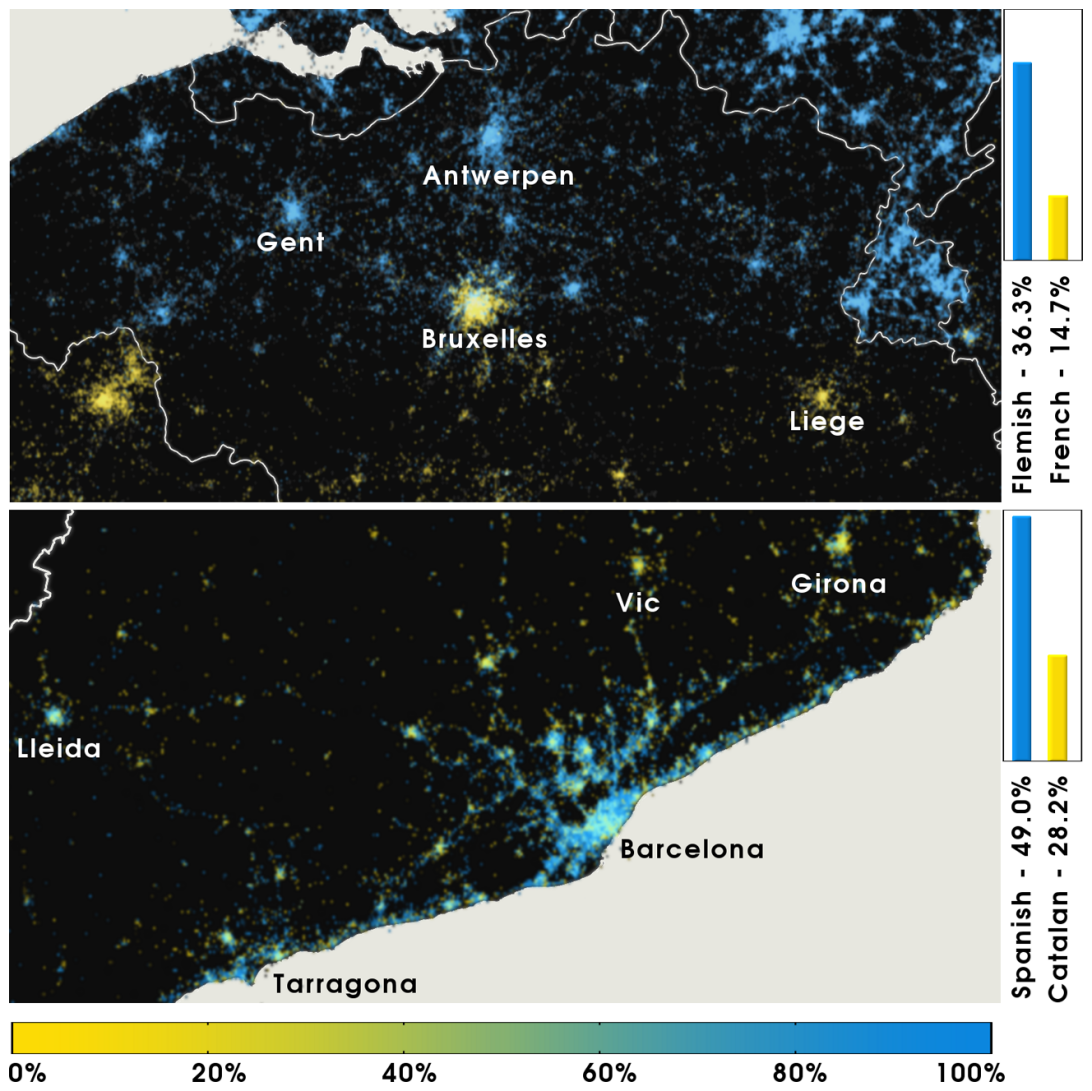
Language polarization in Belgium and Catalonia, Spain. In each cell (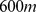 resolution) we compute the user-normalized ratio between the two languages being considered in each case. A) Belgium. B) Catalonia. The color bar is labeled according to the relative dominance of the language denoted by blue. In Belgium, English accounts for 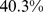 of the language share.

**Regional level**. To understand the geospatial heterogeneity of different linguistic backgrounds, we drill down data to small - within-country- scales. It is interesting, for instance, to look at the spatial distribution of the different languages in multilingual regions. Figure 8-A illustrates the geographical distribution of languages used in Belgium, where the North part of the country uses predominantly Flemish, while in the South of the country the dominant language is (Walloon) French. Overall, Flemish accounts for 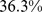 of the users, while French is the language of 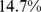 of the users within the country borders, i.e. Dutch is 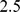 times more popular than French. Census data set the Dutch to French ratio (as first Languages) to 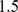 [24]. The result emerging from the Twitter analysis is qualitatively correct, the quantitative mismatch being explained by the different Twitter penetration in neigboring France and Netherlands, whose dominant language is of course French and Dutch. In the first case, the number of users per 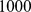 inhabitants is 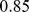, while in the second is 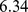, more than 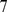 times higher (see also Fig. 2). The Dutch speaking population of Belgium finds itself embedded in a much richer Twitter environment, and consequently is more involved in the microblogging activity.

Moving to a within-country scale, Figure 8-B shows the linguistic distribution in Catalonia, an autonomous region of Spain. Here Catalan and Spanish are clearly intermixed (particularly in Barcelona), even though Spanish is the most popular language, with a share of 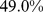 of the users where Catalan represents 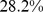 of the signal, making that Spanish 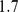 times more popular than Catalan. Interestingly, the Spanish to Catalan ratio is 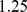 when the habitual language of adults living in Catalonia is considered, according to a survey performed in 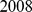 by the Institute of Statistics of Catalonia [25]. In this case the Twitter data is close to the census data, although some considerations are in order. First, census data do not take into account the presence of tourists, whose Twitter activity is on the other hand recorded. Second, Twitter users may be biased towards the most common languages, in order to reach a wider audience. This interpretation is corroborated by the fact that while in our dataset Catalan and Spanish account for the 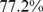 of the users, they represent the habitual language of 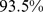 of the population according to the above mentioned survey. In the same way, English, which according to census data is customarily spoken by less than 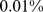 of the resident population, is adopted by 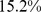 of the users. Going at a deeper level of inspection, we see that the Catalan language is more widely used in the central and Northern part of the region than in the area of Barcelona and the coast connecting this city to Tarragona. Remarkably, this pattern agrees with the overall picture provided by census data [25], thus confirming once again the validity of online data in providing meaningful informations, even at the within-country scale.

**Figure 9:**
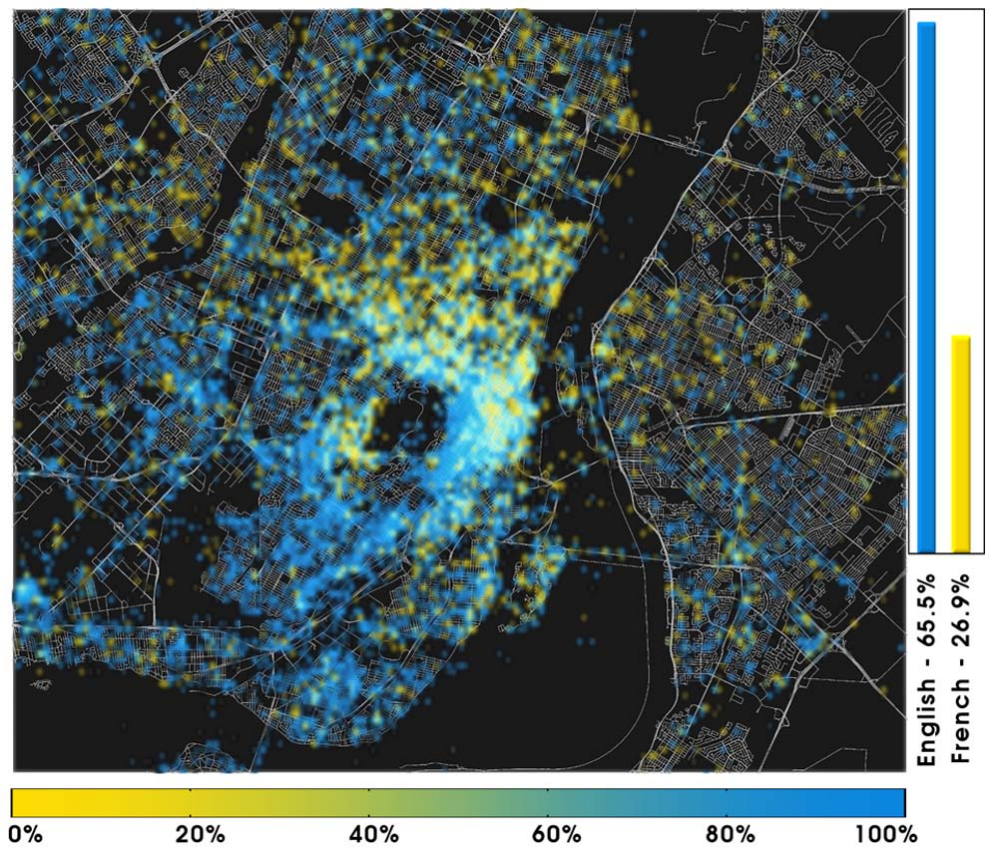
Language polarization in Montreal, QC, Canada. English and French are considered. In each cell (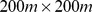) we compute the user-normalized ratio between English and French (excluding all other languages). Blue - English, Yellow - French. The color bar is labeled according to the relative dominance of English to French.

**City level**. The high quality of the GPS geolocalized signal allows the inspection of the language demographics of single cities. Figure 9 shows the city of Montreal, where English and French are the most used languages. While English is significantly more popular (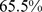 of users, vs. the French 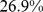), there appear to be spatial segregation, with French being more popular in the northern neighborhoods. Overall, English is 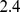 times more popular than French in our signal, while the situation is the opposite according to census data surveying languages spoken at home, where French is 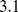 times more frequent than English [26]. This reversal is not easy to interpret, but we speculate that the geographical location of Montreal, and the fact that we do not consider the entire metropolitan population, along with the fact that English is in general the privileged communication language in North America, are two factors that might play an important role.

**Figure 10:**
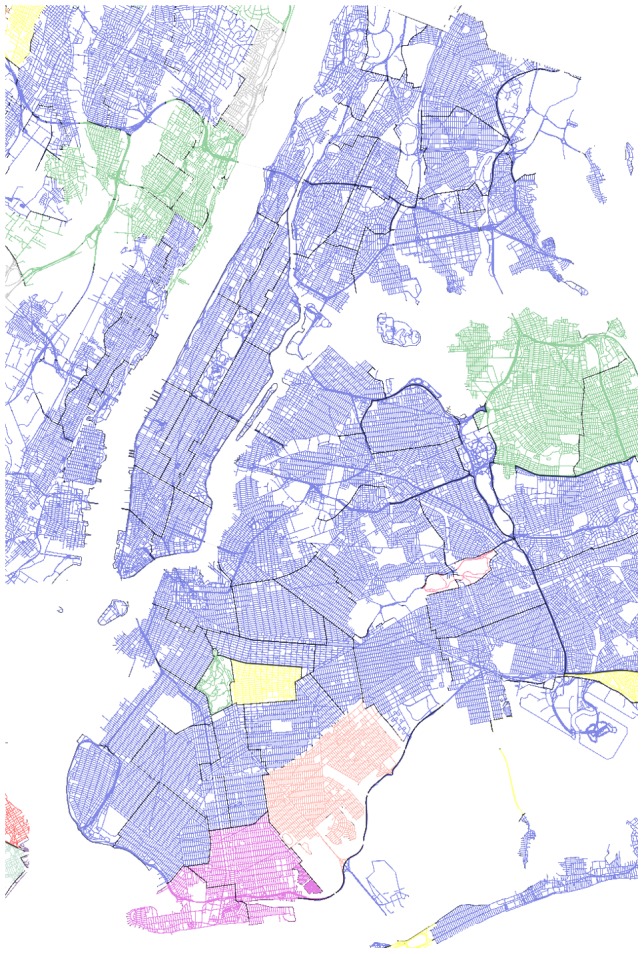
Language polarization in New York City, NY, USA. The second language by district or municipality (in the case of New Jersey state) is shown. Blue - Spanish, Light Green - Korean, Fuchsia - Russian, Red - Portuguese, Yellow - Japanese, Pink - Dutch, Grey - Danish, Coral - Indonesian.

The same analysis can be performed at the level of city neighborhood. In the case of New York City, a city known for its cultural diversity, several non-English speaking communities are already well-defined and documented [27]–[31]. For this case study, we partition NYC, Long Island, and New Jersey state into districts, towns, and municipalities, respectively. We do not consider the signal in English (since it is the official language, and homogeneously predominant in the area) and we focus instead on the language exhibiting the second largest number of users inside each district/town. Some of the most popular communities are those of Spanish speakers in Harlem, Bronx, and parts of Queens [27]. However, Spanish is spoken by people from many different cultural backgrounds and it is also widely used across the United States. It is thus difficult to estimate the exact location and dimensions of these communities solely based on Twitter signal. In fact, it is clear that Spanish dominates as a second language in a number of districts of Figure 10. Remarkable, on the other hand, is the clear delimitation of other communities. The Korean communities in Palisades Park, NJ and Flushing, NY are of considerable size and also very socially active [28], [29]. Marine Park, NY, on the other hand, has a long history of Dutch immigration that dates back to the first European settlers in the area [30]. Another notable example is the case of Coney Island, NY, which is home to the largest Russian community in the United States [31].The high resolution of our dataset allows us to visualize these communities without any a priori assumptions.

### Seasonal variations

**Figure 11:**
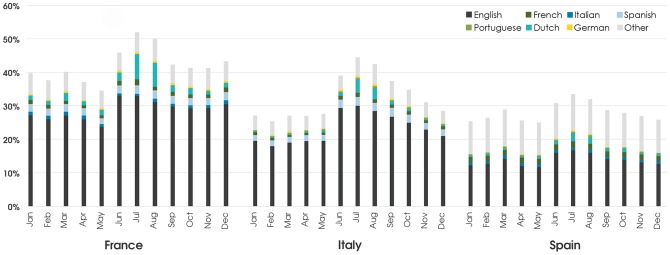
Monthly variations in Language use. Fraction of minority languages in specific countries as a function of the month. Increases in a specific language share indicate the presence of tourists visiting the country. Peaks are clearly visible during the local summer period.

Now that we have a good characterization of the relative linguistic composition of each country we can assess the of use our data to study and analyze seasonal variations of language composition, as this would give us valuable insights onto population movements occurring over the course of a year. In particular, we might expect that during more touristic seasons one could observe a relative decrease in traffic occurring in the local dominant language and a corresponding increase in content being generated in foreign languages. In Fig. 11 we show the relative contributions of minority languages from users within a given country as a function of the month of the year. In particular we single out traditional touristic destinations, such as France, Italy, and Spain, where clear variations are indeed visible during the summer.

Our analysis allows not only to identify the aggregate touristic fluxes, but also to infer the regions of origin on the basis of the observed language. Of course, the patterns we observe are certainly slightly biased by the specificity of our observation point, so that for example the contribution of Dutch is likely to be constantly overestimated due to the high penetration of Twitter in the Netherlands. However, the possibility of observing seasonal fluxes is absolutely remarkable if we consider the low cost, both in terms of time and resources, that a Twitter survey requires, compared to more traditional approaches. Moreover, monitoring social networks allows us to gain a real-time perspective of the fluxes, which is of course extremely hard to achieve through demographic studies.

## Discussion

In this paper we have characterized the worldwide linguistic geography as observed from the Twitter platform, aggregating microblogging data at different scales, from country level down to the neighborhood scale. Although we show that Twitter penetration is highly heterogeneous and closely correlated with GDP, we find that the statistical usage pattern of the microblogging platform turns out to be independent from such factors as country and language. This feature allows us to address different issues, such as linguistic homogeneity at the country level, the geographic distribution of different languages in bilingual regions or cities, and the identification of linguistically specific urban communities. Focusing on specific case-studies, we have shown that while Twitter trends mirror census data quite accurately, specific deviations might emerge when comparing data that can be influenced by the adoption rate of the microblogging platform or the fact that English is the most widely used language in Twitter. This is not surprising, and corroborates previous analysis pointing out the possible distortions induced by observing the World through Twitter [13], [32]. Finally, the analysis of temporal variations of the language composition of a given country opens up the possibility of observing traveling patterns and identifying in real time seasonal traveling and mobility patterns. The presented results confirms the potential and opportunities offered by open access data -such as microblogging posts- in the characterization and analysis of demographic and social phenomena.

## Materials and Methods

### Data Collection

The datased was obtained by extracting tweets from the raw Twitter Gardenhose feed [33]. The Gardenhose is an unbiased sample of 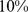 of the entire number of tweets, thus providing a statistically significant real time view of all activity within the Twitter ecosystem [34]. Twitter added support for explicit geotagging of tweets since November 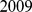, by providing API hooks that could be used by third party developers to embedded GPS coordinates within the metadata of each tweet. Since high quality GPS systems are increasingly common in mobile devices, this feature immediately became popular with mobile application developers and is currently available in hundreds of different Twitter clients. On average, about 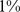 of the tweets contain GPS information. The accuracy of modern GPS technology, as indicated by GPS.gov [35], appears to be as high as just a few meters within 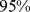 confidence. This resolution is of relevance particularly when investigating heterogeneities at neighborhood level.

### Language Detection

Automatically determining the language in which a certain text was written is problem of great practical importance for machine learning and data mining. Perhaps the better known example of this is a feature in Google's popular web browser, Chrome, that offers to translate a page from it's original language to the users native language has a feature that offers to translate a page to the users preferred language. The library that detects the original language of the page leverages Googles extensive experience with data mining and has been extracted from Chromes source code and made available separately as the “Chromium Compact Language Detector” [36], a library that was extracted from the open source version of Google's Chrome browser that is currently in use by millions of browsers around the world. The Chromium Compact Language Detector library returns a series of candidate languages each with a corresponding probability (e.g., “Spanish 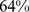, French 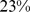, Italian 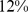, Portuguese 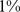
”). To ensure the accuracy of the result, we label only those tweets where the top language is over 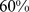 probable, and do not consider the others.

### Geolocalization and Statistics

We restrict our analysis to tweets containing GPS coordinates, i.e. generated by using a smartphone with an Internet connection. This choice allows for the maximum geographical resolution, but inevitably reduces the volume of available signal. In fact, the data we have used for this paper constitutes just about 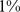 of the signal we have collected, which on its turn is approximately 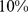 of the total Twitter volume.

The amount of geolocalized tweets could be increased by considering self-reported informations. In fact, users are encouraged to provide their location information in the user profile, but it is not subject to any format restriction. Moreover, Twitter platforms do not prompt the user for an update of this field, thus any change to this metadata field has to be spontaneous and made voluntarily. For this reason, the information in the user profile is sometimes erroneous or has low granularity. While the research community is on a continuous quest to understand how to mine and geocode this data, doing so brings about many challenges [37]. Moreover, when addressing temporal variations in mobility patterns, the use of smartphone GPS coordinates is required.

The metadata accompanying a tweet may also contain the geographical coordinates of a previous location in the field of self-reported location. These `historical' locations might bias statistical measures involving mobility and/or fine graining, thus we considered them only in generating the language maps (Belgium, Catalonia, NYC). All sets of analysis performed at the country level make use solely of live-GPS coordinates. We consider only those countries for which our signal is generated by at least 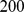 users, normalized by their activity and location. So if a user emits 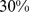 of her tweets from a given country she will contribute as 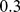 users to that country. 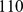 countries satisfy this minimum user threshold.

Finally, it is crucial stressing that every set of statistical measures performed in this paper is done at the user level, in order to reduce the noise that bots or cyborgs might add to the analysis. If not suitably addressed, in fact, their presence could induce wrong conclusions on the day-to-day behavior of the average person [38].

## References

[1] González MC, Hidalgo CA, Barabási AL (2008) Understanding individual human mobility patterns. Nature 453: 779. 18528393 10.1038/nature06958

[2] Onnela JP, Saramaki J, Hyvonen J, Szabo G, Lazer D, et al (2007) Structure and tie strengths in mobile communication networks. Proceedings of the National Academy of Sciences 104: 7332–7336. 10.1073/pnas.0610245104 PMC1863470 17456605

[3] Hale S, Gaffney D, Graham M (2012) Where in the world are you? geolocation and language identification in twitter. Technical report.

[4] Conover M, Ratkiewicz J, Gonçalves B, Haff J, Flammini A, et al. (2011) Predicting the political alignment of twitter users. In: IEEE Third International Conference on Social Computing (SOCIALCOM). p.192.

[5] Sang E, Bos J (2012) Predicting the 2011 dutch senate election results with twitter. EACL 2012: 53.

[6] Gonçcalves B, Perra N, Vespignani A (2011) Modeling users' activity on twitter networks: Validation of dunbar's number. PLoS One 6: e22656. 21826200 10.1371/journal.pone.0022656 PMC3149601

[7] Borge-Holthoefer J, Rivero A, García I, Cauhé E, Ferrer A, et al (2011) Structural and dynamical patterns on online social networks: the spanish may 15th movement as a case study. PLoS One 6: e23883. 21886834 10.1371/journal.pone.0023883 PMC3158778

[8] Tumasjan A, Sprenger T, Sandner P, Welpe I (2010) Predicting elections with twitter: What 140 characters reveal about political sentiment. In: Proceedings of the Fourth International AAAI Conference on Weblogs and Social Media. pp.178–185.

[9] Culotta A (2010) Towards detecting inuenza epidemics by analyzing twitter messages. In: Proceedings of the First Workshop on Social Media Analytics. ACM, pp.115–122.

[10] Salathe M, Khandelwal S (2011) Assessing Vaccination Sentiments with Online Social Media: Implications for Infectious Disease Dynamics and Control. PLoS Computational Biology 7: e1002199. 22022249 10.1371/journal.pcbi.1002199 PMC3192813

[11] Salathe M, Bengtsson L, Bodnar TJ, Brewer DD, Brownstein JS, et al (2012) Digital Epidemiology. PLoS Comput Biol 8: E1002616. 22844241 10.1371/journal.pcbi.1002616 PMC3406005

[12] Kulshrestha J, Kooti F, Nikravesh A, Gummadi K (2012) Geographic dissection of the twitter network. In: In Proceedings of the 6th International AAAI Conference on Weblogs and Social Media (ICWSM).

[13] Mislove A, Lehmann S, Ahn Y, Onnela J, Rosenquist J (2011) Understanding the demographics of twitter users. In: Fifth International AAAI Conference on Weblogs and Social Media.

[14] Hong L, Convertino G, Chi E (2011) Language matters in twitter: A large scale study. In: International AAAI Conference on Weblogs and Social Media. pp.518–521.

[15] Giannotti F, Pedreschi D, Pentland A, Lukowicz P, Kossmann D, et al (2012) A planetary nervous system for social mining and collective awareness. The European Physical Journal Special Topics 214: 49–75.

[16] Williams CH, editor (1988) Language in Geographic Context. Multilingual Matters, Ltd.

[17] Baronchelli A, Loreto V, Tria F (2012) Language dynamics. Advances in Complex Systems 15.

[18] Poblete B, Garcia R, Mendoza M, Jaimes A (2011) Do all birds tweet the same?: characterizing twitter around the world. In: Proceedings of the 20th ACM international conference on Information and knowledge management. ACM, pp. 1025–1030.

[19] Weerkamp W, Carter S, Tsagkias M (2011) How people use twitter in different languages. In: Proceedings of the ACM WebSci'11, June 14-17 2011 ,Koblenz,Germany. p.1.

[20] Takhteyev Y, Gruzd A, Wellman B (2012) Geography of twitter networks. Social Networks 34: 73–81.

[21] Languages of the world. Summary by language size. Available: http://www.ethnologue.org/ethno_docs/distribution.asp?by=size.Accessed 2012 December.

[22] Languages of the world. Summary by language size. Available: http://en.wikipedia.org/wiki/List_of_languages_by_total_number_of_speakers.Accessed 2013 Jaunary.

[23] Mislove A, Lehmann S, Ahn YY, Onnela JP, Rosenquist JN (2011) Understanding the demographics of twitter users. In: In Proceedings of the Fifth International AAAI Conference on Weblogs and Social Media.

[24] Europeans and their languages. Available: http://ec.europa.eu/public_opinion/archives/ebs/ebs_243_en.pdf.Accessed 2012 December.

[25] Usos lingüístics. llengua inicial, d'identificació i habitual. Available: http://www.idescat.cat/dequavi/?TC=444&V0=15&V1=2.Accessed 2012 September.

[26] Population by language spoken most often at home and age groups, 2006 counts, for canada, provinces and territories, and census subdivisions (municipalities) with 5; 000- plus population - 20% sample data. Available: http://www12.statcan.ca/census-recensement/2006/dp-pd/hlt/97-555/T402-eng.cfm?Lang=E&T=402&GH=7&GF=24&G5=1&SC=1&RPP=100&SR=1&S=1&O=D&D1=1.Accessed 2012 December.

[27] Lobo A, Flores R, Salvo J (2002) The impact of hispanic growth on the racial/ethnic composition of new york city neighborhoods. Urban Affairs Review 37: 703–27.

[28] Seoul Mates: Thriving Korean communities make Fort Lee and Palisades Park a boon to epicures. Available: http://njmonthly.com/articles/best-of-Jersey/seoul_mates.html.Accessed 2012 December.

[29] The Korean Community Services Of Metropolitan New York, Inc. Available: http://www.kcsny.org/.Accessed 2012 December.

[30] Marine Park. Available: https://www.nycgovparks.org/parks/marinepark/history.Accessed 2012 December.

[31] Brighton Beach, A Voyage To Russia. Available: http://offmetro.com/ny/2008/04/13/brighton-beach-a-voyage-to-russia/.Accessed 2012 December.

[32] Gayo-Avello D (2012). I wanted to predict elections with twitter and all i got was this lousy paper a balanced survey on election prediction using twitter data.

[33] Ratkiewicz J, Conover M, Meiss M, Gonçcalves B, Patil S, et al.. (2011) Truthy: Mapping the spread of astroturf in microblog streams. Twentieth International World Wide Web Conference 249.

[34] Guide to the Twitter API Part 3 of 3: An Overview of Twitters Streaming API. Available: http://blog.gnip.com/tag/gardenhose/.Accessed 2013 January.

[35] GPS Accuracy. Available: http://www.gps.gov/systems/gps/performance/accuracy/.Accessed 2013 January.

[36] Candless MM (2012). http://code.google.com/p/chromium-compact-language-detector/.

[37] Hecht B, Hong L, Suh B, Chi EH (2011) Tweets from justin bieber's heart: the dynamics of the location field in user profiles. In: Proceedings of the SIGCHI Conference on Human Factors in Computing Systems. New York, NYUSA: ACM, CHI '11, pp.237–46. doi:10.1145/1978942. 1978976. URL http://doi.acm.org/10.1145/1978942.1978976 .

[38] Chu Z, Gianvecchio S, Wang H, Jajodia S (2010) Who is tweeting on twitter: human, bot, or cyborg? In: Proceedings of the 26th Annual Computer Security Applications Conference. New York,NY,USA :ACM, ACSAC '10 , pp.21–30. doi:10.1145/1920261.1920265. URL http://doi. acm.org/10.1145/1920261.1920265.

